# DNA Methylation Level Changes in Transgenic Chinese Cabbage (*Brassica rapa* ssp. *pekinensis*) Plants and Their Effects on Corresponding Gene Expression Patterns

**DOI:** 10.3390/genes12101563

**Published:** 2021-09-30

**Authors:** Jee-Soo Park, Yun-Hee Shin, Young-Doo Park

**Affiliations:** Department of Horticultural Biotechnology, Kyung Hee University, 1732 Deogyoung-daero, Giheung-gu, Yongin-si 17104, Gyeonggi-do, Korea; jeesoo_92@naver.com (J.-S.P.); yunhee94@naver.com (Y.-H.S.)

**Keywords:** bisulfite sequencing, *Brassica rapa*, differentially methylated regions, methylation, transgenic plants

## Abstract

Plant tissue culture is an in vitro technique used to manipulate cells, tissues, or organs, and plays an important role in genetic transformation. However, plants cultured in vitro often exhibit unintended genetic and epigenetic variations. Since it is important to secure the stability of endogenous and exogenous gene expressions in transgenic plants, it is preferable to avoid the occurrence of such variations. In this study, we focused on epigenetic variations, exclusively on methylation level changes of DNA, in transgenic Chinese cabbage (*Brassica rapa* ssp. *pekinensis*) plants. To detect these methylation level changes of DNA, bisulfite sequencing was performed and the obtained sequences were compared with the ‘CT001’ reference genome. Differentially methylated regions (DMRs) of DNA between the non-transgenic and transgenic lines were detected by bisulfite sequencing, and ten DMRs located in exonic regions were identified. The regions with methylation variations that were inherited and consistently maintained in the next generation lines were selected and validated. We also analyzed the relationship between methylation status and expression levels of transformant-conserved DMR (*TCD*) genes by quantitative reverse transcription-PCR. These results suggested that the changes in methylation levels of these DMRs might have been related to the plant transformation process, affecting subsequent gene expression. Our findings can be used in fundamental research on methylation variations in transgenic plants and suggest that these variations affect the expression of the associated genes.

## 1. Introduction

Tissue culture is a widely used tool in plant biotechnology, including *Agrobacterium*-mediated transformation [1]. Transgenic plants are often cultured in vitro and undergo regeneration to produce numerous identical seedlings; hence, the stable expression of the transgene is a critical issue for such plants. However, genetic and epigenetic variations have been reported to occur in transgenic plants and can affect their genomic stability [2,3], thus disrupting the genetic uniformity of these plants. In addition, such variations are inherited. 

Variation derived from tissue cultures, termed somaclonal variation, has been observed in various crops [4]. It has been proposed that alterations in the epigenome may be a cause of somaclonal variation [5,6,7,8,9]. Changes in DNA methylation patterns are frequently observed in regenerated and transgenic plants, and have been suggested to cause phenotypic variation through the modulation of gene expression [10,11]. 

Epigenetics is the study of processes by which a change in the degree of gene expression occurs without a change in the DNA sequence. DNA modification in eukaryotes occurs primarily in cytosine bases, typically by DNA methylation enzymes at the carbon position of cytosine 5. DNA methylation in plants is carried out by a DNA methyltransferase in CG, CHG, and CHH (H = C, A, or T) sequences. A defect in this methyltransferase induced a change in DNA methylation patterns, producing the phenotype of flowering delay in Arabidopsis plants [12]. CG hypomethylation of the *flowering wageningen* (*FWA*) transcription factor partly contributed to expression of the *FWA* gene and delayed flowering in the *met1* mutant. CHG methylation significantly decreased in *chromomethylase 3* (*CMT 3*) Arabidopsis mutants, resulting in a high regenerative capacity [13]. CHG methylation can hence control regenerative capacity and cell potency. 

The level of DNA methylation in plants is dynamically regulated by the interactive reactions of methylation and demethylation. Unlike animals, plants have genes that can directly remove the 5th position of cytosine (5**^m^**C), making them more efficient in DNA demethylation [14]. DNA methylation and demethylation inhibit and induce gene expression, respectively. Furthermore, these processes act as a memory of the patterns of expression [11,12]. 

Specifically, epigenetic variations are mainly expressed as alterations in DNA methylation levels, which can modulate gene expression [15]. DNA methylation changes in the promoter and gene body regions affect the regulation of gene expression and/or function of the protein [4,10,11].

Various methods such as methylation-sensitive amplification polymorphism and methylation-specific PCR (MSP) [16,17,18], have been used to detect methylation variations. Owing to the advent of next-generation sequencing (NGS), DNA methylation changes can be examined genome-wide using diverse methodologies such as bisulfite sequencing (BS-Seq) [19], methylated DNA immunoprecipitation sequencing [20], or bisulfite amplicon sequencing [21].

Chinese cabbage (*Brassica rapa* ssp. *pekinensis*) is one of the most important vegetable crop worldwide. The reference genome of *B. rapa* variety Chiifu-401-42 was published in 2011 [22] and the pseudomolecule genome of the inbred line ‘CT001’ was constructed for genome research [23]. In particular, despite the importance of Chinese cabbage, there were few whole-genome bisulfite sequencing (WGBS) studies on this plant and few studies on transgenic Chinese cabbage plants. 

In this study, we focused exclusively on methylation level changes of DNA in transgenic Chinese cabbage plants developed by *Agrobacterium*–mediated transformation procedure and in vitro culture. WGBS was performed on non-transgenic and transgenic plants derived from the Chinese cabbage inbred line ‘CT001’. DNA methylation patterns of the non-transgenic and transgenic plants were studied, and transgenic plant-specific differentially methylated region (DMR) candidates were selected. Among the conserved DMRs, ten *TCDs* that were located in the exonic region of fully annotated genes and occurred in more than two contexts were also selected. In addition, it was studied whether DNA methylation variations in transgenic plants induced by transformation procedure and in vitro culture were inherited and maintained through generational progression. Finally, the effects of changes in methylation levels on gene expression in transgenic plants were also studied. The findings of this study are expected to provide basic resources on DNA methylation variations in transgenic plants.

## 2. Materials and Methods

### 2.1. Plant Material and Bisulfite Sequencing

To detect methylation variations in transgenic plants, IGA transgenic Chinese cabbage plants with downregulated expression of the glutathione S-transferase gene, generated as described previously [24], were used in this study. Non-transgenic Chinese cabbage (*Brassica rapa* ssp. *pekinensis*) inbred line ‘CT001’ was used as a control line. IGA transgenic plants were developed from inbred line ‘CT001’ through *Agrobacterium*-mediated transformation and cultured in vitro.

The confirmed transgenic lines, IGA7, IGA74, and IGA743 were used to analyze methylation variation. Seeds from the non-transgenic ‘CT001’ and transgenic lines were cultivated for two weeks in a greenhouse at Kyung Hee University (Yongin, Korea). Total genomic DNA was obtained from the leaf tissues of non-transgenic and transgenic lines using the modified sodium dodecyl sulfate method [25]. DNA concentration and quality were measured using the Trinean DropSense instrument (Trinean, Belgium) and PicoGreen assay (Molecular Probes, Eugene, OR, USA). To verify the presence of the T-DNA, PCR and T-DNA inserted site analysis were conducted according to a previously reported study [26].

The quality and quantity of the genomic DNA samples were checked using the PicoGreen assay for WGBS library construction. Agarose gel electrophoresis was also performed to ascertain the quality of the genomic DNA samples. The genomic DNA of each transgenic line was fragmented using a Covaris sonication system (Covaris S2). Following fragmentation, libraries were constructed using the Illumina Nextflex bisulfite-seq kit (Illumina, San Diego, CA, USA). The fragmented DNA was ligated with 5′ and 3′ adaptors, and the adaptor-ligated fragments were amplified and purified. The ligated DNA was bisulfite-converted using the EZ DNA Methylation-Gold kit (Zymo Research, Orange, CA, USA). After bisulfite modification, size selection, PCR amplification, and quality control (QC) of the library were performed. The resultant DNA was quantified using qPCR (Life Technologies, CA, USA), and the insert size was assayed using the Agilent Bioanalyzer 2100 system (Agilent, Santa Clara, CA, USA). The qualified libraries for each sample were sequenced using the HiSeq X system (Illumina). Removal of low-quality read and reads containing adaptor sequences was performed using Trimmomatic software [27].

### 2.2. Sequence DATA Processing and Analysis

Trimmed sequence reads were mapped to ‘CT001’ pseudomolecule reference sequences [23] using Bismark (v0.10.1) under default parameters [28]. Methylation calls were extracted after excluding duplicate sequences. The DNA methylation level was calculated using sites that had more than 95% mapping coverage levels and cytosines covered with an average of nine reads. The outputs were imported to genome browsers, in sequence alignment map (SAM)/binary alignment map (BAM) formats for visualization, and direct exploration.

The ^m^C density and average methylation level of each transgenic line were determined. The ^m^C density refers to the number of cytosine methylations in each sequence context of the aligned reads. In addition, each type of cytosine methylation in the transgenic lines was determined. The average cytosine methylation level was calculated based on the ratio between the number of methylated cytosines and total cytosines within a mapped read present in each transgenic line.

### 2.3. Selection of DMR Candidates

The DNA methylome patterns of the transgenic lines were studied, and transgenic line-specific DMR candidates were selected and analyzed. To identify DMRs across the entire genome of the non-transgenic and transgenic lines, the DSS package (http://bioconductor.org/packages/release/bioc/html/DSS.html; accessed on 31 May 2021) [29] was used in the R environment. Even though, DSS R package does not include false discovery rate (FDR) calculations, FDR was considered and analyzed in DML (differentially methylated locus) analysis, a step before DMR analysis. For DMR analysis, DML analysis should be performed first, and *q* ≤ 0.05 was applied to estimate the locus. Cytosines within methylation loci that presented an average five-fold coverage were used to calculate methylation levels. Regions with a difference of more than 40% in each context compared with the non-transgenic line were defined as DMRs. DMR calling for each transgenic line (IGA7, IGA74, and IGA743) was performed with the callDMR function using all default parameters, except for P-value thresholds of 0.05 and delta values of 0.1. 

The genomic distributions of DMRs in the transgenic lines were investigated. Gene ontology analysis for methylation-related genes was performed using DAVID Bioinformatics Resources v6.8 (http://david.ncifcrf.gov/; accessed on 9 June 2021) [30]. Functional annotation clustering was analyzed based on corresponding TAIR IDs. Classification stringency was set to medium, and those with an enhancement score of more than 0.4 were selected.

In addition, expression analyses of methylation-related genes were performed with the *Arabidopsis* Information Resource (TAIR) ID using the eFP browser (http://bar.utoronto.ca/efp/cgi-bin/efpWeb.cgi; accessed on 21 June 2021). A homology search was performed using basic local alignment search tool (BLAST).

In silico analysis of the DMR candidates was performed using the ‘CT001’ pseudomolecule genome browser. The BAM files of the non-transgenic ‘CT001’, T_1_ (IGA7), T_2_ (IGA74), and T_3_ (IGA743) lines were loaded onto the genome browser. *TCDs* corresponding the condition that DMR was located in the exonic region of the gene and maintained in the next generation were selected. The methylation status of the selected transformant-conserved DMRs (*TCDs*) genes for the CG, CHG, and CHH methylation patterns of each line was compared. The methylation states of CG, CHH, and CHG can be visually verified in silico analysis.

### 2.4. Expression Analysis of Genes Associated with DMR Candidates 

To determine the relationship between DNA methylation status and expression levels of genes within the candidate DMRs in the T_1_ (IGA7), T_2_ (IGA74), and T_3_ (IGA743) lines, quantitative reverse transcription PCR (qRT-PCR) analysis of the selected *TCD* genes was performed. We selected 10 *TCD*s located in the exonic region of annotated genes with different methylation levels in all three transgenic lines. Four individual plants of the T_1_ line (IGA7), three individual plants of the T_2_ line (IGA74), and three individual plants of the T_3_ line (IGA743) were selected for analysis.

Total RNA was isolated from the leaf tissues of the non-transgenic and transgenic lines using the Plant Total RNA Extraction Kit (TaKaRa, Otsu, Japan) in accordance with the manufacturer’s instructions. The qRT-PCR assay was performed using a Roter-Gene^TM^ 6000 (Corbett, Sydney, Australia) and TransStart^®^ Top Green qPCR SuperMix (TransGen Biotech, Beijing, China). The total reaction volume was 20 μL, including 1 μL cDNA, 10 pmol of each primer, and 10 μL 2× QuantiSpeed SYBR mix. The primers used for qRT-PCR are listed in Table S1. The PCR conditions were as follows: pre-denaturation for 10 min at 95 °C, followed by 40 cycles of denaturation for 10 s at 95 °C, annealing and extension for 30 s at 60 °C. Melting curve analysis of the PCR products was performed by increasing the temperature from 60 to 95 °C with a temperature increment rate of 0.1 °C/s. Fluorescence intensity data were collected at the end of each cycle and analyzed using the instrument software. The cycle threshold (Ct) value of each sample was used to calculate the relative gene expression levels via the ΔΔCt method [31]. The actin gene was used as the endogenous housekeeping gene for normalization of the target genes. To ensure the specificity of the results, qRT-PCR analysis was repeated three times, and the average value and standard errors (SE) were analyzed. Standard errors are indicated as bars in Figure 3.

### 2.5. MSP for DMR Candidates

MSP was conducted to examine the CpG islands with changed methylation states of *TCD* genes in non-transgenic ‘CT001’, T_1_ (IGA7), T_2_ (IGA74), T_3_ (IGA743), and T_4_ (IGA7434) lines. Genomic DNA (1 µg) of each line was treated with bisulfite using the EZ DNA Methylation-Gold™ Kit (Zymo research, CA, USA) according to the manufacturer’s instructions. Primers for MSP were designed using MethPrimer 2.0 (Table S2). PCR was performed with 20 μL reaction mixtures using the Maxime™ PCR PreMix Kit (iNtRON, Seongnam, Korea) containing the primer sets designed based on the selected *TCD* genes (Table S2) and bisulfite-treated DNA segments. The MSP conditions were as follows: pre-denaturation for 10 min at 95 °C, followed by 40 cycles of 10 s at 95 °C and 30 s at 60 °C, 30 s at 72 °C, and a final extension for 10 min at 72 °C. PCR amplicons were loaded onto a 1% agarose gel, separated, and observed under ultraviolet light.

## 3. Results

### 3.1. Bisulfite Sequencing and Mapping

Before bisulfite sequencing, we performed a PCR analysis to confirm the insertion of T-DNA, and chromosome number counting to confirm the chromosomal stability of the transgenic lines. WGBS of the non-transgenic and transgenic lines was conducted to determine the DNA methylation status of the transgenic lines. The ambiguously mapped or duplicate reads were removed, and only uniquely mapped reads were retained for further analyses. After trimming the bisulfite sequencing data using Trimmomatic, an average of 8.1 Gb of WGBS data was obtained and the paired mapped reads were mapped to the ‘CT001’ pseudomolecule reference genome using Bismark. Approximately 79.7% of the clean reads could be independently mapped to the reference genome. Thus, an average of six million properly mapped paired reads were retained.

**Figure 1:**
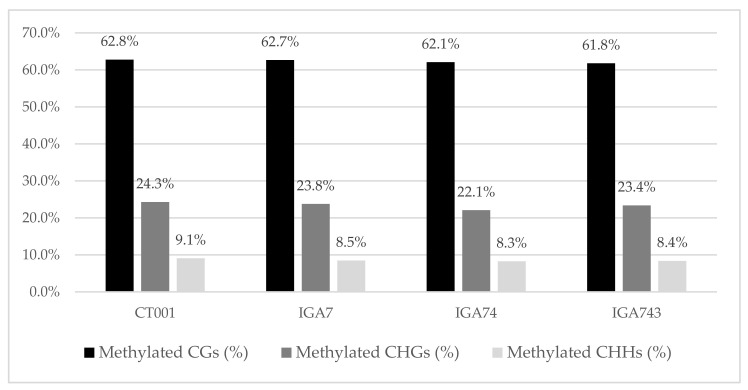
Methylation rates of the non-transgenic and transgenic lines. Proportions of methylated cytosines in the 3 contexts (^m^Cs ≥ 3), classified as CG, CHG, and CHH (H indicates A, C, or T).

The average methylation level in the total genome of each transgenic line was analyzed and was summarized in Table S3. The DMRs in the CpG, CHG, and CHH contexts (where H is any base except G) of each transgenic plant were categorized. The levels of DNA methylation in these three contexts were determined for each region of each transgenic line and compared with that in the non-transgenic control line (Figure 1). DMR calling for transgenic lines resulted in the identification of an average of 39 million methylated CGs (^m^CG) (62.2% of all CGs), 13 million ^m^CHGs (23.1% of all CHGs), and 13 million ^m^CHHs (8.4% of all CHHs). 

### 3.2. Confirmation of Selected TCDs

DMR calling was performed using the WGBS data of each transgenic line, which were compared with those of the non-transgenic line. In addition, genome-wide DNA methylation of each transgenic line was investigated based on the WGBS data of the transformants. As a result, 1642 DMRs, including 1237 DMRs in the CG context, 183 DMRs in the CHG context, and 222 DMRs in the CHH context on the exonic region were identified in the T_1_ transgenic line. Among them, we selected exonic regions with methylation patterns maintained in the T_2_ and T_3_ lines. In particular, two or more overlapping exonic regions were selected and named as conserved DMRs. Finally, we identified 102 conserved DMRs, including 28 DMRs in the CG context, 39 DMRs in the CHG context, and 35 DMRs in the CHH context. The list of genes associated with the DMRs identified in all three transgenic lines is shown in Table S4. 

Conserved DMRs within exonic regions were clustered based on their corresponding TAIR IDs using the DAVID Bioinformatics Resources v6.8 (http://david.ncifcrf.gov/; accessed on 9 June 2021) to investigate the relationship between genes with differences in methylation patterns in the transgenic lines (Table 1). When selecting DMR, T_1_ transgenic line with different methylation status were first compared to non-transgenic control line, CT001 and then DMRs inherited and maintained in next generation lines (T_2_ and T_3_) were selected. Therefore, the number of DMRs was greatly reduced from 1642 to 10. As a result, only a few of categories (Leucine rich repeats) were statistically significant. Functional annotation enriched for conserved DMRs in the transgenic lines showed that they were mainly associated with DNA polymerase activity and signal transduction functions. Functional analysis of the genes within the selected conserved DMRs revealed that these genes were related to diverse functions, including protein phosphorylation and nucleic acid fusion, and production of calcium-dependent lipid-binding protein, reductase thioredoxin family protein, and calcium-binding protein TCH2. 

Among them, DMRs of CT001_A07241320 and CT001_A07421310 appeared in all contexts, whereas DMRs of CT001_A08284340 and CT001_A03126570 appeared in the CHG and CHH contexts. Among the conserved DMRs, ten that were located in the exonic region of fully annotated genes and occurred in more than two contexts were selected and named *TCDs* (Table 2).

**Figure 2:**
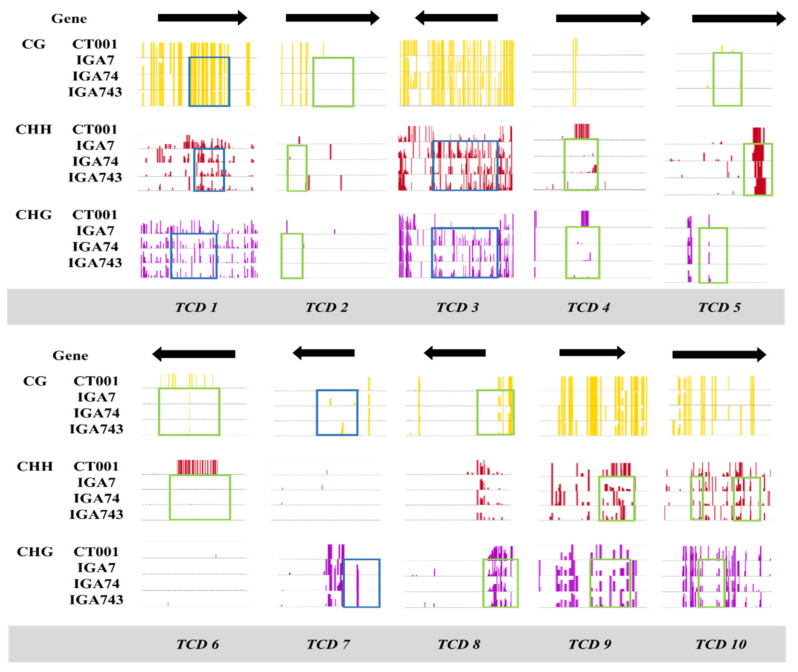
In silico methylation pattern analysis of the transformant-conserved DMRs (TCDs) between the non-transgenic control line ‘CT001’ and transgenic lines (T_1_, T_2_, and T_3_). Black arrows indicate directions of the genes. The green boxes indicate hypomethylated DMRs, and the blue boxes indicate hypermethylated DMRs in the transgenic lines.

For in silico analysis of the selected *TCDs*, the BAM files of the non-transgenic, T_1_, T_2_, and T_3_ transgenic lines were loaded onto the genome browser and the methylation status of their selected *TCD* genes was visualized and compared. Figure 2 summarizes the results of the in silico analysis of the *TCDs*. The CG, CHG, and CHH methylation patterns of each line were compared in parallel. Of the ten selected *TCDs*, seven DMRs were hypomethylated compared with those of the non-transgenic line, whereas three were hypermethylated. 

### 3.3. Correlation between Methylation Status and Expression of TCD Genes 

**Figure 3:**
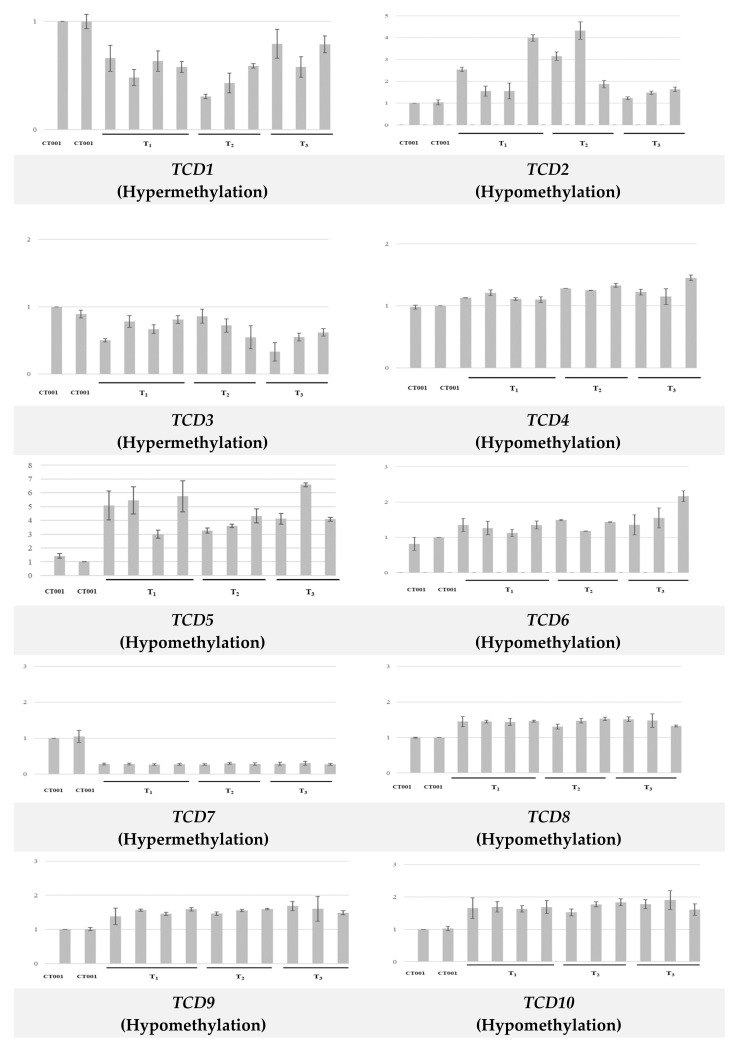
Gene expression analysis of 10 selected genes within transformant-conserved DMRs (TCDs) between the non-transgenic and transgenic lines. Expression levels of each DMR gene in T_1_, T_2_, and T_3_ generations of IGA were compared with inbred line ‘CT001’. *Y*-axis indicates relative mRNA expression, and bars on the graph indicate the SE of the means (*n* = 3).

We analyzed the relationship between gene expression patterns in *TCDs* and methylation patterns of transgenic lines. DMRs identified in all three transgenic lines were selected, and primer sets were designed based on sequences of coding loci identified in transgenic lines to analyze gene expression. To determine whether altered methylation patterns of a DMR affected the expression of the gene within the DMR, total RNA was extracted and cDNA was synthesized for each transgenic line. RT-PCR of the ten selected *TCD* genes was then conducted using cDNA of each transgenic line and primer sets for the selected DMRs, and the amplicon with the expected product size was identified (Figure S1). Gene expression in the non-transgenic and transgenic lines was quantified by qRT-PCR using the same primer sets as those used for RT-PCR. As expected, the gene expression levels positively correlated with the methylation levels. The expression of *TCD* genes with hypermethylated DMR patterns in the transgenic line was downregulated. In contrast, the expression of *TCD* genes with hypomethylated DMR patterns in the transgenic line was upregulated (Figure 3). Among the genes with hypermethylation, the gene expression of *TCD1* in transgenic lines decreased 1.25 to 3.3 times compared with that of non-transgenic line ‘CT001’. In addition, the gene expression of *TCD5* with hypomethylation in the transgenic line increased 3 to 6.5 times compared with that in the non-transgenic line. 

### 3.4. Determination of DNA Methylation Patterns by MSP

**Figure 4:**
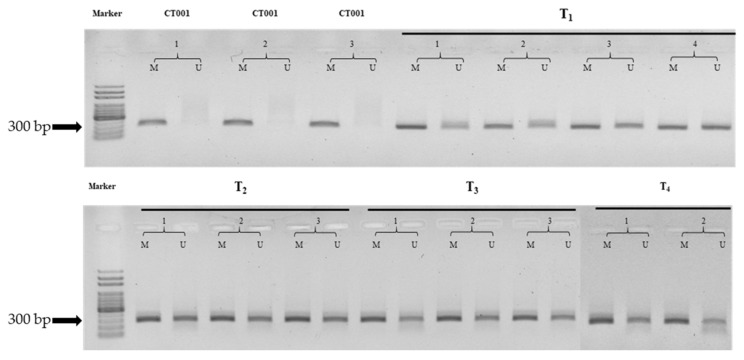
Methylation-specific PCR (MSP) analysis of *TCD8* in the non-transgenic and transgenic lines. MSP was conducted to examine the methylation status of the three CT001, four T_1_ (IGA7), three T_2_ (IGA74), three T_3_ (IGA743) and two T_4_ (IGA7434) lines. M primer set amplified the methylated DNA with 288 bp and the U primer set amplified the unmethylated DNA with 290 bp. CT001, inbred line; T_1_, T_2_, T_3_, and T_4_, transgenic lines of IGA. M, methylated PCR products; U, unmethylated PCR products.

MSP analysis was conducted to visualize the methylation state in non-transgenic and transgenic lines. When MSP was performed using bisulfite-treated DNA (as the template) and primer sets, the M primer set amplified the methylated DNA and the U primer set amplified the unmethylated DNA. Analysis of ten *TCD* genes using the MSP method produced significant results, especially for *TCD8* genes. MSP data of *TCD8* showed that methylated PCR products were present in ‘CT001’, whereas unmethylated PCR products were not (Figure 4). In contrast, methylated and unmethylated regions were visualized using MSP amplicons from the transgenic lines (Figure 4). 

## 4. Discussion

Some of the causes of somaclonal variation in tissue culture are transposable elements, genetic variations such as single nucleotide polymorphisms (SNPs) and insertions/deletions(InDels), and epigenetic variations due to methylation changes. DNA methylation variation is the change in the methylation level of the promoter or exonic region without a change in the DNA sequence. The most common method to detect such changes is to conduct methylome analysis of the genome through WGBS, also known as BS-seq, methyl-seq, or methylC-seq [32,33,34]. Many studies have reported WGBS of various crops under abiotic and biotic stress. WGBS of regenerated plants has also been studied, but most of these plants were sourced from food crops, including maize and rice [35,36,37,38]. Despite the importance of Chinese cabbage, there have not been many WGBS studies on this plant, and few studies have been conducted on transgenic plants [39].

In this study, WGBS of transgenic lines was carried out to confirm the DNA methylation patterns and gene expression levels. Approximately 79.7% of the reads mapped to the reference genome suggested that we had obtained fundamental data that could enable meaningful analysis. The average percentages of methylation of CG, CHG, and CHH contexts in the transgenic lines were 62.2%, 23.1%, and 8.4%, respectively, which were much higher than those in *Arabidopsis thaliana* (24%, 6.7%, and 1.7% for CG, CHG, and CHH, respectively) [32]. The percentage of methylation of CG sequences in vertebrates, including zebrafish and mice, is known to account for nearly 80% of all methylation. In contrast, all types of methylation occur in plants. CG is the most methylated, CHG is moderately methylated, and CHH is methylated at the lowest level in plants [40]. From an evolutionary perspective, plants belonging to the same genus have similar methylation patterns. For example, an investigation of the methylation patterns of the DNA methyltransferase, *CMT 3*, in *Brassica rapa* and *Brassica oleracea* indicated that the distribution patterns of the three contexts were similar [41,42]. 

To examine the influence of the *Agrobacterium*-mediated transformation process on DNA methylation status, we identified the DMRs in transgenic lines through in silico analysis using a genome browser. Based on these data, the expression levels of the *TCD* genes were determined by qRT-PCR. The gene expression level was positively associated with the methylation level. 

In this study, methylation differences were observed not only in the 1kb-up promoter region of genes but also in the exon and intron. Changes in methylation status in the promoter and coding regions can affect gene expression, however, the analysis was conducted focusing on exonic region.

By examining the function of the conserved DMR-related genes, we studied the possible correlation between the transformation process and changes in methylation patterns. The leucine-rich repeat (LRR) is used as a module for the interaction of many plant proteins [43]. Changes in the methylation level of LRR partially affect the transmission of external signals to inside the cell. It is engaged in common biological processes, including defense and response to abiotic and biotic stresses. In addition, genes involved in membrane components and membrane transport were clustered. In particular, ten *TCDs* existing in exons, and duplicated in two or more contexts, were selected and further analyzed based on their TAIR IDs. Genes with transport-, transferase-, and plasma membrane-related functions were mainly clustered. 

*TCD1* and *TCD2* are genes with different DNA methylation levels in all contexts, however, they do not have corresponding Brassica or TAIR IDs and their functional annotations are understudied. The *TCD3* gene showed different DNA methylation patterns in CHG and CHH contexts, and was found to be similar to AT3G47200. However, its exact function is not known. *TCD4* is expected to function as a gene associated with the RNA export factor, *silencing defective 5* (*SDE5*). *SDE5* affects DNA methylation by regulating RNA-directed DNA methylation (RdDM) [35]. *TCD5* is a gene encoding the F-box / kelch-repeat protein, and *TCD6* is associated with *tyrosine-sulfated glycopeptide receptor 1* (*PSY1R*). *PSY1R* is not only involved in growth and development, but also in plant defense [44]. *PSY1*-reactive genes encode genes localized in the cell wall that regulate carboxylesterase activity, whereas differentially expressed genes in *psy1r* mutant plants mainly localize to the nucleus through molecular functions of ion binding and activity of transcription factors [45]. 

*TCD7* is a G-box binding factor, and GF14 omega encodes a 14-3-3 protein. According to previous studies, 14-3-3 proteins interact with *abscisic acid response-binding factor 3* (*ABFBI3*) to regulate the basic regulation/leucine zipper (bZIP) transcription factor, *ABA insensitive 5* (*ABI5*). *ABI5* is known to confer resistance to salt stress upon Arabidopsis plants [46,47,48]. *TCD8* encodes *domains rearranged methyltransferase 3* (*DRM3*). *DRM3*, which is homologous to *DRM2*, is known to regulate RdDM in Arabidopsis plants, and has been reported to be involved in the regulation of DNA methylation [49,50]. We also confirmed the results of a previous study, which showed that *DRM3* regulated DNA methylation by enhancing the transcriptional elongation of RNA polymerase V or stabilizing RNA polymerase V transcripts [51]. Based on studies that analyzed the function of *DRM3*, it was assumed that the DMR of the gene equivalent to *TCD8* was related to epigenetic changes such as DNA methylation in response to external stresses occurring during various processes of transformation. *TCD9* encodes *potassium uptake transporter 1* (*AtKUP1*) and is involved in K+ transport in plants. The sodium–potassium pump is a membrane protein present in cell membranes that functions as an enzyme for the hydrolysis of adenosine triphosphate (ATP). It consumes the energy generated by decomposing ATP, moving three sodium ions out of the cell and two potassium ions into the cell. It is known to generate and maintain electrical and concentration gradients, and prevent the cell volume from growing. The *AtKUP1* gene has been reported to be highly expressed in plants exposed to salt stress [52]. *TCD10* encodes *chromosome transmission fidelity 7* (*CTF7*), which is homologous to the *establishment of cohesion 1* (*Eco1*) gene. *Eco1* enables sister chromatid cohesion, which is essential for cell division. The transgenic line inhibited the expression of the *CTF7* gene in the Arabidopsis plants that showed decreased development of dwarfism, anthers, and infertility, and was closely linked to the DNA repair process and cell division [53,54]. Based on our results, it was assumed that the methylation pattern of the *TCD10* gene was hypomethylated, and that the expression of the gene changed to cope with problems such as external environmental changes or DNA anomalies during the process of recovery.

Among the seven annotated genes, *TCD7* only showed a hypermethylation pattern. *TCD7* matched with AT1G78300, which encodes a 14-3-3 protein that play significant roles in the regulation of plant responses to abiotic stresses, including drought, temperature, and salinity, and biotic stresses, including plant hormones and exotic pathogens. The 14-3-3 genes exhibit various levels of up- or down-regulation [55]. Therefore, it can be assumed that the expression of the AT1G78300 gene decreases in response to stress. The decrease in gene expression of *TCD7* due to hypermethylation was predicted to defend against abiotic and biotic stresses generated during transformation. Seven out of the ten genes identified were analyzed using the eFP browser. The expression levels of the genes tended to increase by 2–4 times on average during the callus induction process; therefore, these genes were considered to be involved in the process of transformation and in vitro culture. In particular, the expression level of galactose oxidase/kelch repeat superfamily protein (AT1G67480, *TCD5*) increased 20-fold after callus induction, and it was probably associated with the regeneration process. Compared with those of ‘CT001’, the genes were hypomethylated, suggesting that they were expressed more during regeneration.

These results suggested that the changes in methylation levels of these DMRs might have been related to the *Agrobacterium*–mediated transformation process, affecting subsequent gene expression. Furthermore, the generated DMRs were inherited and maintained as the generations progressed. MSP analysis methods made it possible to visualize the methylation patterns and confirmed that these changed patterns were inherited to next generations. *TCD8* was found to be methylated in the non-transgenic line and partially unmethylated in the T_1_ transgenic line, and this partially unmethylated state was maintained in the T_2_, T_3_, and T_4_ transgenic lines (Figure 4). These results show that the *TCD8* gene is methylated in the general condition of the *B*. *rapa* plant, but it is unmethylated under various environmental stresses through de-differentiation and re-differentiation during the transformation process. This change in methylation patterns was also maintained in the T_2_, T_3_, and T_4_ generations. 

It may be considered that transgenes may have indirectly influenced DNA methylation. However, in this study, IGA transgenic lines with downregulated expression of the glutathione S-transferase gene [24] were used to examine the methylation level changes of DNA in transgenic plants. IGA transgenic lines were developed through *Agrobacterium*-mediated transformation and cultured in vitro. The glutathione S-transferase gene is involved in the synthesis of isothiocyanates in Chinese cabbage, but not in the methylation system. By down-regulating this gene, phenylethylisothiocyanate can be accumulated in cells [24]. In addition, it was also confirmed that single copy of transgene was inserted into the intergenic region of the IGA genome [26]. Therefore, the presence of a transgene with down-regulating vector of glutathione S-transferase gene was not expected to be a major factor for methylation changes. 

In conclusion, the results of this study demonstrate that changes of methylation status can be assumed to be induced during *Agrobacterium*–mediated transformation and in vitro tissue culture, and subsequently affect their gene expression. And these changes of methylation levels can be inherited through generation progression. 

## 5. Conclusions

Methylation level changes have been observed in transgenic plants cultured in vitro. These undesired changes in DNA methylation pose a problem for the genetic stability of transgenic plants. In this study, BS-Seq was conducted using non-transgenic and transgenic lines of the Chinese cabbage inbred line ‘CT001’. Methylation variations in the transgenic lines and their effects on gene expression were analyzed. Consistently identified DMRs in T_1_, T_2_, and T_3_ lines were detected, and ten DMRs located in the exonic regions of a gene were selected and analyzed. The findings of this study will help understand the variation in methylation in transgenic plants and can be applied in further research.

## Figures and Tables

**Table 1:** Functional annotation clustering of the conserved DMRs in the transgenic lines.

Cluster Enrichment Score	Category ^z^	Description	Count	*p* Value
1.45	INTERPRO	Leucine-rich repeat, typical subtype	4	3.1 × 10^−3^
	SMART	LRR TYP	4	5.3 × 10^−1^
	INTERPRO	Leucine-rich repeat	4	2.3 × 10^−2^
	INTERPRO	Leucine-rich repeat-containing N-terminal, type 2	4	5.0 × 10^−2^
	UP_KEYWORDS	Receptor	4	6.2 × 10^−2^
	UP_KEYWORDS	Leucine-rich repeat	4	2.3 × 10^−1^
	GOTERM_MF_DIRECT	Kinase activity	4	2.6 × 10^−1^
0.43	GOTERM_CC_DIRECT	Integral component of membrane	11	3.0 × 10^−1^
	UP_SEQ_FEATURE	Transmembrane region	5	3.2 × 10^−1^
	UP_KEYWORDS	Transport	5	3.7 × 10^−1^
	UP_KEYWORDS	Transmembrane helix	11	4.0 × 10^−1^
	UP_KEYWORDS	Transmembrane	11	4.1 × 10^−1^
	UP_KEYWORDS	Membrane	12	4.8 × 10^−1^
0.42	UP_KEYWORDS	Nucleotide-binding	8	6.2 × 10^−2^
	UP_KEYWORDS	ATP-binding	6	2.0 × 10^−1^
	UP_KEYWORDS	Serine/threonine-protein kinase	3	3.3 × 10^−1^
	GOTERM_MF_DIRECT	ATP binding	6	3.8 × 10^−1^
	INTERPRO	Serine/threonine-protein kinase, active site	3	4.0 × 10^−1^
	GOTERM_MF_DIRECT	Protein serine/threonine kinase activity	3	4.0 × 10^−1^
	SMART	S TKc	3	5.2 × 10^−1^
	INTERPRO	Protein kinase, catalytic domain	3	5.3 × 10^−1^
	INTERPRO	Protein kinase-like domain	3	5.5 × 10^−1^
	INTERPRO	P-loop containing nucleoside triphosphate hydrolase	3	5.7 × 10^−1^
	UP_KEYWORDS	Transferase	6	5.9 × 10^−1^
	UP_KEYWORDS	Kinase	3	6.2 × 10^−1^

^z^ The terms derived from reference databases. INTERPRO, terms from InterPro protein database; SMART, analysis of domain architectures; UP_KEYWORDS, keywords from UniProtKB; GOTERM_MF, Gene ontology term of molecular function; GOTERM_CC, Gene ontology term for cellular component; UP_SEQ_FEATURE, Uniprot Sequence Feature.

**Table 2:** Information of genes within the transfomant-conserved DMRs (TCDs) in the transgenic lines.

Name	Context	Methyl in Transformants ^Z^	Gene ID	Brassica ID	TAIR ID	Description
*TCD 1*	CG CHG CHH	Hyper	CT001_A07241320	-	-	-
*TCD 2*	CG CHG CHH	Hypo	CT001_A07421310	-	-	-
*TCD 3*	CHG CHH	Hyper	CT001_A08284340	Bra034540	-	-
*TCD 4*	CHG CHH	Hypo	CT001_A05184770	Bra027247	AT3G15390	Putative nuclear RNA export factor SDE5 isoform X1
*TCD 5*	CHG CHH	Hypo	CT001_A07260420	Bra004227	AT1G67480	F-box/kelch-repeat protein
*TCD 6*	CG CHH	Hypo	CT001_A07264360	Bra016068	AT1G72300	Tyrosine-sulfated glycopeptide receptor 1
*TCD 7*	CG CHG	Hyper	CT001_A07243410	Bra012325	AT1G78300	G-box binding factor GF14 omega encoding a 14-3-3 protein
*TCD 8*	CG CHG	Hypo	CT001_A09347510	Bra031188	AT3G17310	Probable inactive DNA (cytosine-5)-methyltransferase DRM3
*TCD 9*	CHH CHG	Hypo	CT001_A01011320	Bra013553	AT2G30070	High affinity potassium transporter
*TCD 10*	CHG CHH	Hypo	CT001_A03126570	Bra024010	AT4G31400	Protein chromosome transmission fidelity 7

^z^ Methylation status of transgenic lines compared to non-transgenic line. Hyper, hypermethylation; Hypo, hypomethylation.

## Data Availability

The obtained sequencing data (5 fastq files) generated during the current study were deposited in the National Agricultural Biotechnology Information Center (NABIC) (http://nabic.rda.go.kr, accessed on 21 June 2021) in Rural Development Administration (RDA), South Korea with the following accession numbers (CT001 Re-seq: NN-1902, CT001 Bs-seq: NN-6312, IGA7 Bs-seq: NN-6314, IGA74 Bs-seq: NN-7496, IGA743 Bs-seq: NN-6315). The sequencing data is publicly available.

## Supplementary Materials

The following are available online at https://www.mdpi.com/article/10.3390/genes12101563/s1, Figure S1. RT-PCR analysis of the non-transgenic and transgenic lines using primer sets for transformant-conserved DMRs (TCDs). Table S1. List of primer sets for quantitative RT-PCR analysis. Table S2. List of primer sets for methylation-specific PCR analysis. Table S3. Average methylation level in total genome of the non-transgenic and transgenic lines. Table S4. List of genes within the conserved DMRs identified in the transgenic lines.
