# Supplementary material for: DNA Methylation Level Changes in Transgenic Chinese Cabbage (Brassica rapa ssp. pekinensis) Plants and Their Effects on Corresponding Gene Expression Patterns
Source: Genes (Basel). 2021 Sep 30;12(10):1563. doi: 10.3390/genes12101563 (PMC8535332; doi:10.3390/genes12101563)
Supplement: Supplementary file 1 [file genes-12-01563-s001.zip › genes-1351714-supplementary.pdf]

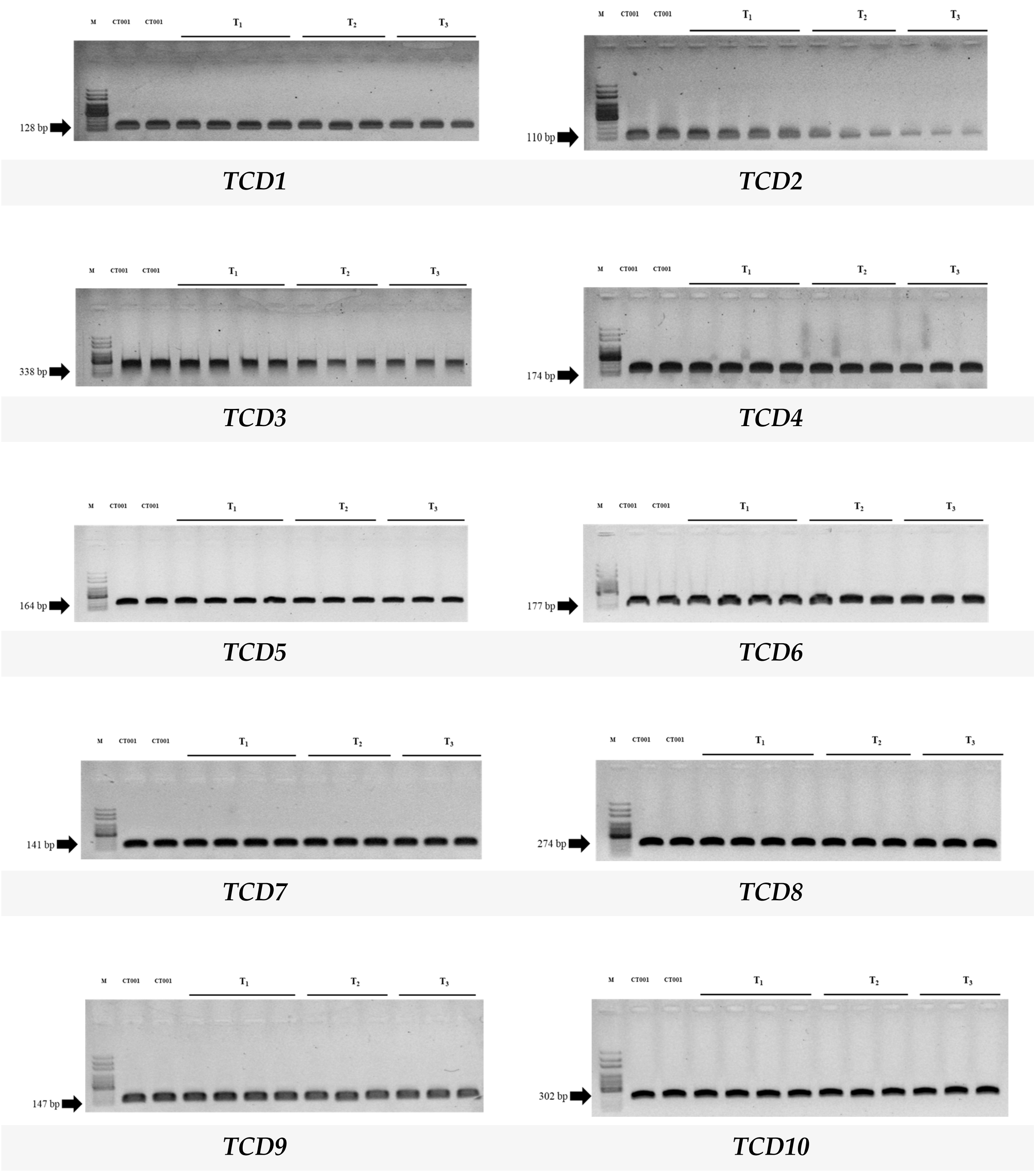

Figure S1. RT-PCR analysis of the non-transgenic and transgenic lines using primer sets for transformant-conserved DMRs (*TCDs*).

Table S1. List of primer sets for quantitative RT-PCR analysis.

Name	Primer	Sequence (5'→3')	Expected product size (bp)
TCD 1	F[z]	GAA CAC GGG TGA TGG AGA T	128
	R	ACC TCA TAC CTG CCA AAC G	
TCD 2	F	AAC TCA TCA GAA ATC CTC CTC	110
	R	AGA CGG CGA GTT TGA GAG A	
TCD 3	F	CTC CAC CAA CAA GTC TCC	338
	R	GGA GAC TTG TTG GTG GAG	
TCD 4	F	ATG CCT GAA TCA AAT GCT CGC	174
	R	GCC TTC TCC GAG CAT CTT AG	
TCD 5	F	GAT GTG TAT AAC CCT CAG TGC	164
	R	GCC ACC ACT TCC CAA GTT TC	
TCD 6	F	GAG GTC CAG AAT GCT GAG G	177
	R	GGT AGA CTA ATC AAT CTG CGC	
TCD 7	F	CAC AAT CGA GGA GCG AAA C	141
	R	TCA CGG ATC GTT TTG ACG C	
TCD 8	F	AGG AGG AGG TGG TGG AGA TT	274
	R	CGC CTT TGT GAA TAC CGT TT	
TCD 9	F	TTG GGA AAG TCT TAC GCA AAG	147
	R	CCC AAC TTG AAG CAG AGA AG	
TCD 10	F	GTT TAG CCG CGT GGG AGA AA	302
	R	CTT GCT GAG ATT CTT GGA TCC	

[Z]F, forward primer; R, reverse primer

Table S2. List of primer sets for methylation-specific PCR analysis.

Name	Primer	Sequence (5'→3')	Expected product size (bp)
**MSP *TCD 8***	MF[z]	TAT TAA ATA GAG AAT ATT AGG TCG A	288
	MR	ATA CTT CGT AAT AAC ACT AAA CGA C	
	UF[y]	TTA TTA AAT AGA GAA TAT TAG GTT GA	290
	UR	AAT ACT TCA TAA TAA CAC TAA ACA AC	

[z]M, forward and reverse primers of methylated DNA; [y]U, forward and reverse primers of unmethylated DNA

Table S3. Average methylation level in total genome of the non-transgenic and transgenic lines.

	Context	CpG		CHG		CHH	
	Name	Total methylated Cs	Relative ratio of ^{m}Cs (%)	Total methylated Cs	Relative ratio of ^{m}Cs (%)	Total methylated Cs	Relative ratio of ^{m}Cs (%)
Control	CT001	38,834,362	62.80%	13,158,821	24.30%	14,228,984	9.10%
T_1	IGA7	36,018,799	62.70%	11,907,922	23.80%	12,110,178	8.50%
T_2	IGA74	40,956,444	62.10%	12,547,429	22.10%	13,253,347	8.30%
T_3	IGA743	39,537,761	61.80%	13,144,114	23.40%	13,603,249	8.40%

Table S4. List of genes within the conserved DMRs identified in the transgenic lines.

CG	CHG	CHH
• CT001_A01003170	• CT001_A01003170	• CT001_A01011320
• CT001_A01003180	• CT001_A01003180	• CT001_A01017400
• CT001_A01023540	• CT001_A01011320	• CT001_A01027200
• CT001_A01028720	• CT001_A01017400	• CT001_A02036620
• CT001_A01035730	• CT001_A01023540	• CT001_A02052780
• CT001_A02052780	• CT001_A01027200	• CT001_A02058860
• CT001_A02058860	• CT001_A01028720	• CT001_A02065390
• CT001_A02062200	• CT001_A01035730	• CT001_A03104360
• CT001_A03084970	• CT001_A02036620	• CT001_A03126570
• CT001_A03399340	• CT001_A02062200	• CT001_A03397530
• CT001_A04142210	• CT001_A02065390	• CT001_A04145490
• CT001_A05162850	• CT001_A03084970	• CT001_A04404580
• CT001_A05175920	• CT001_A03104360	• CT001_A05182060
• CT001_A06416690	• CT001_A03126570	• CT001_A05184770
• CT001_A07241320	• CT001_A03397530	• CT001_A06219970
• CT001_A07243410	• CT001_A03399340	• CT001_A06225040
• CT001_A07246130	• CT001_A04142210	• CT001_A06416690
• CT001_A07264360	• CT001_A04145490	• CT001_A07241320
• CT001_A07265300	• CT001_A04404580	• CT001_A07246130
• CT001_A07421310	• CT001_A05162850	• CT001_A07260420
• CT001_A08299270	• CT001_A05175920	• CT001_A07264360
• CT001_A09332120	• CT001_A05182060	• CT001_A07265300
• CT001_A09344220	• CT001_A05184770	• CT001_A07421310
• CT001_A09347510	• CT001_A06219970	• CT001_A08271920
• CT001_A10366390	• CT001_A06225040	• CT001_A08273660
• CT001_A10379400	• CT001_A07241320	• CT001_A08280970
• CT001_A10381040	• CT001_A07243410	• CT001_A08284340
• CT001_A10384790	• CT001_A07260420	• CT001_A08299270
	• CT001_A07421310	• CT001_A09317820
	• CT001_A08271920	• CT001_A09327490
	• CT001_A08273660	• CT001_A09332120
	• CT001_A08280970	• CT001_A09343100
	• CT001_A08284340	• CT001_A10366390
	• CT001_A09317820	• CT001_A10379400
	• CT001_A09327490	• CT001_A10381040
	• CT001_A09343100	
	• CT001_A09344220	
	• CT001_A09347510	
	• CT001_A10384790	
